# Inflammatory, Insulinemic, and Plant-Based Dietary Patterns and Diabetes Among Patients with Acute Pancreatitis: A Cross-Sectional Analysis of the DREAM Study

**DOI:** 10.3390/nu18213676

**Published:** 2026-11-06

**Authors:** Djibril M. Ba, Tian Qiu, Ni Shi, Nazia Raja-Khan, Qi Sun, Kristen M. Roberts, Anna E. Phillips, Cemal Yazici, Dhiraj Yadav, Marina Basina, Melena Bellin, James L. Buxbaum, Anna Casu, Daisy Duan, Kathleen Dungan, Carmella Evans-Molina, Evan L. Fogel, Chris E. Forsmark, Mark O. Goodarzi, Steven Hughes, Rajesh N. Keswani, Brian T. Layden, Sandra Lord, Stephen J. Pandol, Georgios I. Papachristou, Walter G. Park, Richard Pratley, Vikesh K. Singh, Cate Speake, Guru Trikudanathan, Temel Tirkes, Frederico G. S. Toledo, Vernon M. Chinchilli, Fred K. Tabung, Phil A. Hart

**Affiliations:** 1 Department of Public Health Sciences, Penn State College of Medicine, Hershey, PA 17033, USAvchinchilli@pennstatehealth.psu.edu (V.M.C.); 2 Comprehensive Cancer Center, The Ohio State University Wexner Medical Center, Columbus, OH 43210, USA; 3 Division of Endocrinology, Diabetes and Metabolism, Department of Medicine, Penn State College of Medicine, Hershey, PA 17033, USA; 4 Departments of Nutrition and Epidemiology, Harvard T.H. Chan School of Public Health, Channing Division of Network Medicine, Brigham and Women’s Hospital and Harvard Medical School, Boston, MA 02115, USA; 5 Division of Medical Dietetics, School of Health and Rehabilitation Sciences, The Ohio State University Wexner Medical Center, Columbus, OH 43210, USA; 6 Division of Gastroenterology, Hepatology and Nutrition, Department of Medicine, University of Pittsburgh Medical Center (UPMC), Pittsburgh, PA 15213, USA; 7 Division of Gastroenterology and Hepatology, Department of Medicine, University of Illinois Chicago and Jesse Brown VA Medical Center, Chicago, IL 60612, USA; cyazic2@uic.edu; 8 Division of Endocrinology, Department of Medicine, Stanford University, Stanford, CA 94305, USA; 9 Department of Pediatrics, University of Minnesota Medical School, Minneapolis, MN 55455, USA; bell0130@umn.edu; 10 Division of Gastroenterology, Department of Medicine, Keck School of Medicine of the University of Southern California, Los Angeles, CA 90033, USA; 11 AdventHealth Translational Research Institute, Orlando, FL 32804, USA; anna.casu@adventhealth.com (A.C.);; 12 Division of Endocrinology, Diabetes and Metabolism, Johns Hopkins University School of Medicine, Baltimore, MD 21205, USA; 13 Division of Endocrinology, Diabetes and Metabolism, Department of Internal Medicine, The Ohio State University Wexner Medical Center, Columbus, OH 43210, USA; 14 Department of Pediatrics, Center for Diabetes and Metabolic Diseases, Herman B Wells Center for Pediatric Research, Indiana University School of Medicine, Indianapolis, IN 46202, USA; 15 Division of Gastroenterology and Hepatology, Department of Medicine, Indiana University School of Medicine, Indianapolis, IN 46202, USA; 16 Division of Gastroenterology, Hepatology and Nutrition, University of Florida, Gainesville, FL 32610, USA; 17 Division of Endocrinology, Diabetes and Metabolism, Department of Medicine, Cedars-Sinai Medical Center, Los Angeles, CA 90048, USA; 18 Division of Gastroenterology and Hepatology, Department of Medicine, Northwestern University, Lake Forest, IL 60045, USA; 19 Department of Medicine, University of Illinois Chicago and Jesse Brown VA Medical Center, Chicago, IL 60612, USA; 20 Center for Interventional Immunology, Benaroya Research Institute at Virginia Mason, Seattle, WA 98101, USA; slord@benaroyaresearch.org (S.L.);; 21 Division of Digestive and Liver Diseases, Department of Medicine, Cedars-Sinai Medical Center, Los Angeles, CA 90048, USA; 22 Division of Gastroenterology, Hepatology, and Nutrition, College of Medicine, The Ohio State University Wexner Medical Center, Columbus, OH 43210, USA; 23 Division of Gastroenterology and Hepatology, Stanford University, Stanford, CA 94305, USA; 24 Division of Gastroenterology, Johns Hopkins Medical Institutions, Baltimore, MD 21205, USA; 25 Division of Gastroenterology, Hepatology and Nutrition, Department of Medicine, University of Minnesota Medical Center, Minneapolis, MN 55455, USA; 26 Department of Radiology and Imaging Sciences, Indiana University School of Medicine, Indianapolis, IN 46202, USA; 27 Division of Endocrinology and Metabolism, Department of Medicine, University of Pittsburgh, Pittsburgh, PA 15261, USA; 28 Division of Medical Oncology, Department of Internal Medicine, The Ohio State University Wexner Medical Center, Columbus, OH 43210, USA

**Keywords:** DREAM study, dietary patterns, diabetes

## Abstract

**Background/Objectives:** Plant-based diets are widely recommended for lowering the risk of type 2 diabetes, with the caveat that their health benefits depend on the quality of the foods consumed. This analysis examined associations between five dietary patterns and pre-existing diabetes among patients with acute pancreatitis (AP), a population in which the relationship between diet and diabetes has not been well characterized. **Methods**: Participants were drawn from the Diabetes RElated to Acute Pancreatitis and its Mechanisms (DREAM) study, an ongoing multicenter study of adults aged 18 to 75 years with AP in the U.S. This cross-sectional analysis evaluated five dietary patterns using food frequency questionnaires (FFQ): the reverse Empirical Dietary Index for Hyperinsulinemia (rEDIH), the reverse Empirical Dietary Inflammatory Pattern (rEDIP), the overall plant-based diet index (PDI), the healthful plant-based diet index (hPDI), and the unhealthful plant-based diet index (uPDI). We used multivariable logistic regression models to estimate odds ratios (ORs) for pre-existing diabetes (i.e., prior to the AP episode). **Results:** A total of 647 participants (mean age 49.1 ± 14.5 years) were included. In multivariable-adjusted analyses, participants in the highest versus lowest tertile of PDI had 55% lower odds of pre-existing diabetes (adjusted OR: 0.45; 95% CI: 0.25–0.80; *p*-trend = 0.005). No statistically significant associations were detected for rEDIP, rEDIH, hPDI, or uPDI. **Conclusions:** Higher overall PDI scores were associated with lower odds of prevalent diabetes. Among patients with AP, further studies are needed to evaluate dietary contributions to future diabetes development following AP and identify potential dietary strategies for diabetes prevention.

## 1. Introduction

Dietary patterns characterized by higher consumption of antioxidant- and anti-inflammatory-rich foods, such as plant-based and Mediterranean diets that emphasize fruits, vegetables, legumes, and whole grains, have been associated with a lower risk of diabetes in the general population [1,2]. Conversely, proinflammatory dietary patterns, including those with higher empirical dietary inflammatory index and empirical dietary insulinemic index scores, typically reflect greater consumption of red and processed meats, refined grains, sugar-sweetened beverages, and other ultra-processed foods and have been associated with an increased risk of diabetes in the general population [3,4].

Acute pancreatitis (AP) is a common inflammatory disease of the pancreas, and pre-existing diabetes is frequently present among patients with AP [5,6]. However, evidence examining the connection between dietary patterns and diabetes in patients with AP remains limited. In a cross-sectional study of 108 individuals with AP, higher intakes of fiber, vegetables, and nuts were associated with lower fasting plasma glucose and HbA1c concentrations [7]. Similarly, in our previous analysis from the Diabetes RElated to Acute Pancreatitis and its Mechanisms (DREAM) study, higher intake of the Alternate Mediterranean Diet and the Mediterranean-DASH Intervention for Neurodegenerative Delay (MIND) diet was associated with lower odds of pre-existing diabetes [8]. These observations suggest that dietary patterns rich in antioxidant and anti-inflammatory foods may be associated with favorable glucose metabolism among patients with AP. However, given the cross-sectional design of the present study, the observed associations cannot establish temporal or causal relationships between selected dietary patterns and pre-existing diabetes among patients with AP in the DREAM study. A previous randomized clinical trial suggested that a plant-based dietary intervention improved β-cell function and insulin resistance in overweight adults [9]. Whether these dietary patterns are similarly associated with diabetes after pancreatic injury remains unclear. To our knowledge, no studies have examined the associations of the reverse Empirical Dietary Index for Hyperinsulinemia (rEDIH), the reverse Empirical Dietary Inflammatory Pattern (rEDIP), or plant-based dietary patterns with pre-existing diabetes among patients with AP. This represents an important knowledge gap because AP is characterized by pancreatic injury, inflammation, and metabolic disturbances that may influence glucose homeostasis. Dietary indices capturing complementary aspects of diet, including insulinemic potential, inflammatory potential, and plant-based dietary patterns, may provide insights into the associations between diet and pre-existing diabetes among patients with AP [10,11]. Dietary patterns with higher inflammatory and insulinemic potential may be associated with less favorable metabolic profiles, whereas plant-based dietary patterns characterized by greater fiber intake and antioxidant-rich foods may be associated with more favorable glucose metabolism [10,11,12]. These potential associations may reflect the interplay among systemic inflammation, insulin resistance, and pancreatic exocrine dysfunction in the setting of AP, all of which may contribute to impaired glucose metabolism [10,13,14]. Examining these dietary indices may therefore help characterize the relationships between different dietary patterns and pre-existing diabetes in this high-risk population. The plant-based diet indices (PDI) further distinguish overall plant food consumption from the consumption of plant foods categorized according to their nutritional quality, with the healthful PDI (hPDI) emphasizing healthful plant foods and the unhealthful PDI (uPDI) emphasizing less healthful plant foods. Building on our previous DREAM study of overall diet quality, including the Mediterranean diet and Healthy Eating Index [8], the current analysis focuses on dietary patterns that capture complementary metabolic, inflammatory, and dietary quality dimensions. Specifically, we examined the associations of five dietary indices with pre-existing diabetes among patients presenting with AP in the DREAM study: the rEDIH, the rEDIP, the overall PDI, the hPDI, and the uPDI.

## 2. Materials and Methods

### 2.1. Study Design and Study Population

Participants for this analysis were drawn from the DREAM study (NCT05197920), an ongoing multicenter cohort study of patients with AP in the U.S. [15]. Details regarding the study objectives, design, and assessments have been previously described [15]. In brief, the study aims to determine the incidence and underlying mechanisms of diabetes that develop following an episode of AP. We invited patients with AP who had diabetes before enrollment to complete a baseline assessment without further follow-up. For this cross-sectional analysis, we included participants regardless of diabetes status and used data from the enrollment visit.

The present analysis included 867 participants enrolled as of 21 April 2025. All DREAM study participants were invited to complete a dietary assessment at enrollment using the computer-administered VioScreen Food Frequency Questionnaire (FFQ) [16]. The VioScreen FFQ assesses usual dietary intake over the preceding 90 days. Participants were excluded from the present analysis if they did not complete the FFQ (n = 123), completed it more than 90 days after enrollment (n = 2), were older than 75 years at the time of AP diagnosis (n = 1), or reported extreme daily energy intake (<800 or >4200 kcal for men; <500 or >3500 kcal for women) (n = 94).

### 2.2. Dietary Scores Computation

VioScreen is a self-administered, web- and mobile-based FFQ that includes more than 1500 food images to help respondents identify foods they consume and report consumption frequency and portion sizes [16]. VioScreen’s dietary analysis uses food and nutrient data derived from the University of Minnesota Nutrition Coordinating Center (NCC) Food and Nutrient Database [17]. In the ongoing DREAM study, dietary intake is assessed at baseline and at the 3- and 12-month study visits. For the present analysis, we used baseline dietary data, which captured dietary intake over the preceding 90 days of AP diagnosis. Data on food consumption frequency and portion sizes were used to calculate the dietary scores examined in this study.

The overall PDI assesses adherence to plant-based dietary patterns and includes three main variants: the Overall PDI, healthy (h)PDI, and unhealthy (u)PDI indices. It is based on 18 food components [12,18]. These are grouped into healthy plant foods (whole grains, fruits, vegetables, nuts, legumes, vegetable oils, tea/coffee), less healthy plant foods (fruit juices, sugar-sweetened beverages, refined grains, potatoes, sweets/desserts), and animal foods (animal fats, dairy products, eggs, fish and seafood, meat [including poultry and red meat], and other miscellaneous animal-derived foods) (Supplementary Table S1) [12,18]. We calculated dietary scores from FFQ data by assigning reported food items to predefined food groups, following methods used in prior epidemiological studies [8,12,18]. We categorized intake within each food group into quintiles and assigned each quintile a score from 1 to 5 [12,18]. For the overall PDI, all plant food groups are positively scored, with participants in the highest intake quintile assigned a score of 5, whereas animal food groups are reverse-scored so that higher intake corresponds to lower scores. In the hPDI, healthy plant foods are positively scored, and all other food groups are negatively scored. The uPDI applies an inverted scoring scheme, with less healthy plant foods positively scored and healthy plant and animal foods negatively scored [12,18]. Greater scores indicate higher adherence to the respective diet pattern [12,18].

The Empirical Dietary Index for Hyperinsulinemia (EDIH) evaluates the insulinemic potential of diet from 18 food components associated with c-peptide [3]. The 18 food components included predominantly low insulinemic potential (wine, coffee, whole fruits, whole-milk dairy, green leafy vegetables) and predominantly high insulinemic potential (red meat, regular sodas, diet sodas, cream soups, processed meat, margarine, poultry, butter, French fries, other fish, tomatoes, low-fat dairy, and eggs) (Supplementary Table S2) [3]. We applied a standardized, harmonized method using the Nutrition Data System for Research (NDSR) to calculate EDIH [17]. In brief, we mapped each food intake (g/day) in the FFQ to 168 standardized NDSR food groups and converted it to servings per 100 g using matrix multiplication, with serving sizes based on NDSR definitions from the Dietary Guidelines for Americans (DGA) [19]. The EDIH food group components are weighted by regression coefficients and summed, with scores on a continuum ranging from maximally low-insulinemic to maximally high-insulinemic [3].

The Empirical Dietary Inflammatory Pattern (EDIP) evaluates the pro-inflammatory potential of diet from 18 food components associated with inflammation biomarkers interleukin-6 (IL-6), *C*-reactive protein (CRP), and tumor necrosis factor-α receptor 2 (TNFαR2) [20]. EDIP food components included nine pro-inflammatory groups (red meat, processed meat, organ meat, non-oily fish, low-energy sugary beverages, regular sodas, diet sodas, refined grain, other vegetables, and tomato), and nine anti-inflammatory groups (wine, beer, tea, green leafy vegetables, dark yellow vegetables, fruit juice, snack, and pizza) (Supplementary Table S2). We calculated EDIP using the same standardized methods as EDIH. rEDIH and rEDIP are the reverse versions of the original EDIH and EDIP scores, designed to facilitate comparison with PDI scores and interpretation of results. Higher rEDIH and rEDIP scores indicate greater adherence to dietary patterns associated with lower insulinemic and inflammatory potential, respectively.

### 2.3. Ascertainment of Pre-Existing Diabetes

In this analysis, the presence of diabetes prior to the qualifying AP episode for DREAM enrollment was the dependent variable, defined as any of the following: a prior physician diagnosis of diabetes, use of antidiabetic medications to treat diabetes, or a hemoglobin A1c (HbA1c) level ≥ 6.5% prior to the onset of the AP episode that qualified the participant for study inclusion. Research teams obtained this information through retrospective reviews of electronic medical records at the study site and, when needed, outside records.

### 2.4. Covariates Assessment

Study teams obtained clinical history through medical record abstraction and direct participant interviews. Potential confounders were selected as a priori based on prior literature and clinical expertise regarding factors associated with both diabetes and AP. These included age, sex, race/ethnicity, body mass index (BMI), smoking status, Charlson Comorbidity Index (CCI), hyperlipidemia, prior history of AP, AP etiology and severity, metabolic dysfunction associated with fatty liver disease (MAFLD), hypertension, total caloric intake (assessed via an FFQ), and time from AP diagnosis to FFQ completion. We included biological sex (male/female) as a binary variable. For this analysis, we used a modified CCI that excluded diabetes, the outcome of interest, as well as hypertension and MAFLD, which were assessed separately in the multivariable models. We also included education, annual income, employment status, food insecurity, socioeconomic status index, and social determinants of health in the descriptive characteristics presented in Table 1. However, we excluded these variables from the multivariable regression models because of substantial missing data. The etiology of AP was obtained from a standardized study questionnaire. The etiology was adjudicated by an expert gastroenterologist at each clinical center. For statistical analysis, AP etiology was categorized as gallstones, alcohol, others, or missing. The “other” category included idiopathic, hypertriglyceridemia-induced, drug-induced, genetic, and other etiologies of AP, which were grouped together because of the small sample sizes in each individual category.

### 2.5. Statistical Analysis

We described baseline characteristics by tertile for each dietary index. Univariable analyses summarized categorical variables as frequencies (%) and continuous variables as means (SD). We also generated a correlation matrix to estimate Pearson correlation coefficients between each of the five dietary indices, BMI, and total calorie intake. We adjusted all dietary scores for total energy intake using the residual method [20]. We used multivariable logistic regression models to estimate odds ratios (ORs) and 95% confidence intervals (CIs) for the associations between each dietary index and the odds of pre-existing diabetes, using the lowest dietary index tertile (unhealthy diet) as the reference group. Regression analyses were also conducted with dietary patterns modeled as continuous variables to examine the association between a 1-SD increment in dietary pattern scores and the odds of pre-existing diabetes. We calculated *p* values for linear trends across tertiles by assigning each tertile’s median value to all participants in that tertile and modeling this variable continuously. We used sequential multivariable nested modeling methods to assess the association of interest. Model 1, in addition to total energy, was adjusted for age, sex, and race. Model 2 additionally adjusted for comorbidities, including Charlson Comorbidity Index, hyperlipidemia, hypertension, and MAFLD. Model 3 further adjusted for lifestyle and disease-related factors, including smoking status, history of prior AP, etiology and severity of AP, and time from diagnosis to FFQ completion. Model 4 additionally adjusted for BMI. We included BMI in a separate extended model because it may act as a confounder and/or lie on the potential causal pathway. We assessed the goodness-of-fit of the multivariable logistic regression models using the Hosmer–Lemeshow test. To assess the robustness of the primary findings, we conducted a sensitivity analysis restricted to participants with AP and no prior history of AP to minimize potential misclassification of diabetes etiology from participants with recurrent episodes of AP who may have pancreatogenic diabetes. We also conducted a separate sensitivity analysis in which we imputed missing covariate values by assigning a separate “missing” category for categorical variables and the sample mean for continuous variables. We used SAS (version 9.4; SAS Institute Inc., Cary, NC, USA) to process and harmonize the data and R (version 4.5.1; R Foundation for Statistical Computing, Vienna, Austria) to conduct statistical analyses. All statistical tests were two-sided, and *p* values < 0.05 were considered statistically significant.

## 3. Results

After applying the selection criteria, 647 DREAM participants (mean (SD) age 49.1 (14.5) years) were included in the analysis, of whom 19.6% had pre-existing diabetes at the time of AP diagnosis and qualified for participation in DREAM (Table 1).

Percentages for non-missing categories were calculated among participants with available data, whereas percentages for missing data were calculated using the total number of participants in each tertile. Therefore, percentages may not sum to 100% across all categories. Participants with higher PDI scores were more likely to be younger, non-smokers, have a lower BMI, and have a history of gallstones or prior AP, but were less likely to have MAFLD, hyperlipidemia, or pre-existing diabetes (Table 1). In contrast, participants with higher rEDIP and rEDIH scores were more likely to be older, White, and non-smokers, and less likely to have gallstones, a prior history of AP, hyperlipidemia, or pre-existing diabetes (Table 1). Participants with higher hPDI and uPDI scores tended to be younger, female, White, non-smokers, and have a history of hyperlipidemia. Those with higher hPDI scores also had lower BMI. Thus, higher scores on both indices reflected a mixed dietary pattern, characterized by greater consumption of both healthy and unhealthy plant foods (Supplementary Table S3).

Pearson correlation coefficients among dietary scores ranged from moderate inverse to moderate positive correlations (r = −0.51 to 0.63). The strongest positive correlation was observed between rEDIH and rEDIP (r = 0.63; 95% CI: 0.58–0.68; *p* < 0.001), while the strongest inverse correlation was observed between hPDI and uPDI (r = −0.51; 95% CI: −0.57 to −0.45; *p* < 0.001). Correlations between BMI and dietary scores were weak and not statistically significant (Supplementary Figure S1).

In multivariable-adjusted analyses including BMI, participants in the highest tertile compared with the lowest tertile had 55% lower odds of pre-existing diabetes for overall PDI (adjusted OR: 0.45; 95% CI: 0.25–0.80; *p* for trend = 0.005). In models including PDI dietary scores as a continuous variable, a 1-SD increment in the PDI dietary score was associated with lower odds of pre-existing diabetes (adjusted OR: 0.94; 95% CI: 0.90–0.98) (Table 2).

Compared with the lowest tertile of rEDIP, the highest tertile was not associated with pre-existing diabetes (Model 4, adjusted OR: 0.69; 95% CI: 0.39–1.23; *p* for trend = 0.196). Similarly, no association was observed for rEDIP modeled continuously (per 1-SD increase: adjusted OR: 0.84; 95% CI: 0.62–1.15) (Table 2). For rEDIH, the highest tertile was also not associated with pre-existing diabetes (adjusted OR: 0.92; 95% CI: 0.51–1.64; *p* for trend = 0.814), and the continuous model showed no association per 1-SD increase (adjusted OR: 0.87; 95% CI: 0.66–1.14) (Table 2). Similarly, no statistically significant associations were detected between hPDI and pre-existing diabetes (adjusted OR: 0.68, 95% CI: 0.37–1.22) or between uPDI and pre-existing diabetes (adjusted OR: 0.81, 95% CI: 0.44–1.47) (Supplementary Table S4).

Findings from the sensitivity analysis restricted to participants without a prior history of AP were consistent with those of the primary analysis. Compared with participants in the lowest tertile of PDI, those in the highest tertile had lower odds of pre-existing diabetes (Model 4, adjusted OR: 0.45; 95% CI: 0.21–0.94; *p* for trend = 0.033). No significant associations were observed between pre-existing diabetes and rEDIP, rEDIH, hPDI, or uPDI scores (Supplementary Table S5). In addition, sensitivity analyses incorporating imputed values for missing covariates yielded results consistent with the primary analysis for PDI, with associations remaining materially unchanged (Supplementary Table S6).

## 4. Discussion

Using validated FFQ-based dietary data from a large U.S. study of adults with AP, we found that higher overall PDI scores were associated with lower odds of prevalent diabetes. This association was robust and persisted after adjustment for sociodemographic factors, lifestyle factors (including BMI), comorbidities, and dietary factors, as well as in a sensitivity analysis limited to participants enrolled after their first lifetime AP episode. This observation aligns with broader research showing that plant-based diets are associated with decreased risk of type 2 diabetes in the general population. The observed association between the overall PDI and pre-existing diabetes warrants careful interpretation considering the absence of an association with hPDI. The overall PDI does not always indicate a better or healthier dietary pattern since, in contrast to the hPDI, it assigns good scores to all plant foods, regardless of their nutritional quality or benefit. The differing findings might be due to variations in the dietary components that these indices capture, but our findings do not identify the components that could be responsible for the observed association. Moreover, the absence of a significant association with the hPDI should not be taken as evidence that the associations for the two indices differ. Given the cross-sectional nature of the study and the timing of dietary assessment relative to AP diagnosis at enrollment and pre-existing diabetes, these findings should be interpreted as associations rather than evidence of a protective or harmful effect of either dietary pattern. Additional research is needed to better understand the temporal relationships between diet and diabetes in patients with AP.

For our analysis, we selected five dietary patterns based on their established relationships with insulin sensitivity, impaired glucose oxidation, adiposity, systemic inflammation, and overall dietary quality [10,11,21]. Evidence from prior studies suggests that plant-based dietary patterns are associated with improved glycemic outcomes and a lower risk of type 2 diabetes. Meta-analyses of randomized controlled trials report modest improvements in HbA1c with vegetarian or plant-based interventions, while large prospective cohorts, including the Nurses’ Health Studies, the Health Professionals Follow-up Study, and the Atherosclerosis Risk in Communities (ARIC) study, consistently show lower risk of incident diabetes with higher adherence to plant-based diets [12,22,23,24,25]. Importantly, these findings are primarily based on results from general population cohorts. It is important to generate evidence in an enriched population of AP due to unique factors, including the high frequency of persistent symptoms, risk for nutritional compromise, and likely contributions to diabetes development from pancreatic injury and other mechanisms [5,26,27].

Currently, there are no evidence-based dietary recommendations specifically for diabetes prevention or management among individuals with AP [28]. Generating these recommendations requires data that can temporally link diet patterns with future diabetes development. Thus, the current cross-sectional design does not allow us to conclude definitively that a plant-based diet can prevent diabetes development in AP patients. To the best of our knowledge, this is the first study to simultaneously investigate multiple plant-based dietary patterns and dietary-related inflammatory and insulinemic indices in relation to pre-existing diabetes among adults with AP while accounting for major potential confounding factors. Although we found no significant associations for hPDI, uPDI, rEDIP, or rEDIH, these findings add to the limited evidence on dietary profiles and diabetes in this high-risk population and highlight important areas for future investigation, particularly regarding plant-based diets.

Several biologically plausible mechanisms may help contextualize the potential relationship between overall plant-based dietary patterns and diabetes, although causal relationships cannot be established in this cross-sectional study [29,30,31]. One potential mechanism involves reduced oxidative stress, which occurs when free radicals outweigh the antioxidant defense system [30,31,32]. Reactive oxygen species can be neutralized by a variety of antioxidants found in diets high in fruits, vegetables, whole grains, legumes, nuts, and seeds [33]. By helping to limit oxidative damage to lipids, proteins, and deoxyribonucleic acid (DNA), these dietary components may contribute to maintaining redox balance [30,31,34]. However, because the overall PDI assigns positive scores to all plant foods regardless of their nutritional quality, these mechanisms may not fully explain the observed association with pre-existing diabetes. Furthermore, given the timing of dietary collection relative to diabetes diagnosis, we cannot determine whether the reported dietary patterns preceded diabetes onset or reflect dietary changes following the AP diagnosis.

Our study has limitations to consider when interpreting these results. First, because of high missingness for variables, such as the socioeconomic status index, education, and social determinants of health, we could not include them in the multivariable logistic regression models. Second, because of the cross-sectional design, we cannot rule out reverse causation. Dietary history was collected at the enrollment visit (i.e., following AP diagnosis), while diabetes preceded AP onset. However, the FFQ assessed dietary intake during the 90 days preceding the AP episode, providing information on dietary habits before the acute event. Nevertheless, reported dietary habits may still have been influenced by the AP episode, pre-existing diabetes, or both. Consequently, for the present study, we cannot establish whether the reported dietary patterns preceded the onset of diabetes or contributed to its development, and the observed associations should not be interpreted as causal. Nevertheless, the design allows us to generate hypotheses for future testing in participants with incident diabetes following dietary assessment. Third, as in any observational study, residual confounding may persist despite adjustment for a wide range of potential confounders because of unmeasured factors and potential measurement error in assessed covariates. Fourth, individuals who follow healthy dietary patterns may also engage in other health-promoting behaviors, such as physical activity, which the present analysis did not account for. Future studies should consider incorporating these factors. Fifth, due to the small sample size, less prevalent etiologies of AP were grouped into a single “Others” category, limiting our ability to analyze individual uncommon causes, such as hypercalcemia-induced and drug-induced AP, separately. Although AP etiology was adjusted for in the multivariable regression models, the possibility of residual confounding by specific underlying etiologies cannot be ruled out. Additionally, the current dataset does not allow us to fully assess the diabetes subtypes of included participants with AP. Final analysis following completion of the DREAM study will allow us to more accurately determine diabetes subtypes by incorporating results from autoantibody testing, metabolic testing, and prospective determination of incident cases of DM onset following AP [35]. Lastly, despite using an FFQ, dietary data remain subject to recall bias inherent in self-reported intake measures.

## 5. Conclusions

Using detailed data from the multicenter DREAM study, we found that higher overall plant-based dietary pattern scores were associated with lower odds of pre-existing diabetes among adults with AP. These findings support future prospective investigations of plant-based dietary patterns, as well as other patterns, in relation to diabetes risk following AP. This evidence base is needed to guide dietary modifications following AP to improve metabolic health.

## Figures and Tables

**Table 1:** Characteristics of DREAM participants according to tertile categories of PDI, rEDIP, and rEDIH scores. Higher scores indicate greater adherence to diet patterns.

	PDI			rEDIP			rEDIH		
Characteristics	Tertile 1 N = 214	Tertile 2 N = 219	Tertile 3 N = 214	Tertile 1 N = 215	Tertile 2 N = 216	Tertile 3 N = 216	Tertile 1 N = 215	Tertile 2 N = 216	Tertile 3 N = 216
Scores, median (range)	53.8	60.0	65.9	−0.95	−0.30	0.20	−1.75	−0.93	−0.32
	(42.8, 57.2)	(57.2, 62.7)	(62.7, 80.0)	(−3.31, −0.57)	(−0.57, −0.06)	(−0.06, 2.79)	(−4.75, −1.23)	(−1.23, −0.65)	(−0.65, 0.99)
Age, mean (SD), years	49.1 (14.4)	49.3 (14.6)	48.7 (14.5)	48.9 (14.1)	47.8 (14.8)	50.6 (14.5)	48.1 (13.7)	48.7 (15.3)	50.4 (14.4)
Age group, n (%)									
18–30	25 (11.7)	22 (10.0)	30 (14.0)	23 (10.7)	29 (13.4)	25 (11.6)	25 (11.6)	28 (13.0)	24 (11.1)
31–60	134 (62.6)	134 (61.2)	130 (60.7)	140 (65.1)	133 (61.6)	125 (57.9)	141 (65.6)	125 (57.9)	132 (61.1)
61–75	55 (25.7)	63 (28.8)	54 (25.2)	52 (24.2)	54 (25.0)	66 (30.6)	49 (22.8)	63 (29.2)	60 (27.8)
Sex, n (%)									
Male	93 (43.5)	105 (47.9)	99 (46.3)	115 (53.5)	84 (38.9)	98 (45.4)	126 (58.6)	95 (44.0)	76 (35.2)
Female	121(56.5)	114 (52.1)	115 (53.7)	100 (46.5)	132 (61.1)	118 (54.6)	89 (41.4)	121 (56.0)	140 (64.8)
Race, n (%)									
White/Caucasian	170 (79.4)	166 (76.9)	155 (73.5)	155 (72.8)	158 (73.8)	178 (83.2)	165 (77.5)	166 (77.2)	160 (75.1)
Black	32 (15.0)	34 (15.7)	30 (14.2)	33 (15.5)	42 (19.6)	21 (9.8)	32 (15.0)	33 (15.3)	31 (14.6)
Others, multiple	12 (5.6)	16 (7.4)	26 (12.3)	25 (11.7)	14 (6.5)	15 (7.0)	16 (7.5)	16 (7.4)	22 (10.3)
Missing	0 (0.0)	3 (1.4)	3 (1.4)	2 (0.9)	2 (0.9)	2 (0.9)	2 (0.9)	1 (0.5)	3 (1.4)
Education, n (%)									
Less than high school	8 (4.9)	7 (4.1)	8 (5.1)	12 (7.5)	6 (4.0)	5 (2.8)	8 (4.9)	6 (3.8)	9 (5.4)
High school graduate or equivalent	36 (22.2)	31 (18.3)	30 (19.1)	35 (22.0)	32 (21.2)	30 (16.9)	30 (18.4)	30 (19.1)	37 (22.0)
Some college or associate degree	50 (30.9)	66 (39.1)	46 (29.3)	60 (37.7)	49 (32.5)	53 (29.8)	61 (37.4)	50 (31.8)	51 (30.4)
College graduate or higher	68 (42.0)	65 (38.5)	73 (46.5)	52 (32.7)	64 (42.4)	90 (50.6)	64 (39.3)	71 (45.2)	71 (42.3)
Missing	52 (24.3)	50 (22.8)	57 (26.6)	56 (26.0)	65 (30.1)	38 (17.6)	52 (24.2)	59 (27.3)	48 (22.2)
Annual income, n (%)									
Under $50,000	43 (31.4)	42 (29.0)	44 (31.4)	40 (29.9)	56 (41.8)	33 (21.4)	44 (30.6)	42 (30.7)	43 (30.5)
$50,000–$99,999	37 (27.0)	46 (31.7)	30 (21.4)	46 (34.3)	28 (20.9)	39 (25.3)	41 (28.5)	38 (27.7)	34 (24.1)
Over $100,000	57 (41.6)	57 (39.3)	66 (47.1)	48 (35.8)	50 (37.3)	82 (53.2)	59 (41.0)	57 (41.6)	64 (45.4)
Missing	77 (36.0)	74 (33.8)	74 (34.6)	81 (37.7)	82 (38.0)	62 (28.7)	71 (33.0)	79 (36.6)	75 (34.7)
Employment, n (%)									
Employed	97 (61.4)	108 (65.1)	101 (64.7)	101 (65.2)	91 (61.1)	114 (64.8)	108 (68.4)	97 (61.4)	101 (61.6)
Unemployed for other reasons	44 (27.8)	43 (25.9)	44 (28.2)	37 (23.9)	38 (25.5)	56 (31.8)	38 (24.1)	45 (28.5)	48 (29.3)
Unemployed for medical reasons	17 (10.8)	15 (9.0)	11 (7.1)	17 (11.0)	20 (13.4)	6 (3.4)	12 (7.6)	16 (10.1)	15 (9.1)
Missing	56 (26.2)	53 (24.2)	58 (27.1)	60 (27.9)	67 (31.0)	40 (18.5)	57 (26.5)	58 (26.9)	52 (24.1)
Smoking status, n (%)									
Never smoker	122 (57.3)	120 (55.0)	143 (67.5)	120 (55.8)	129 (60.6)	136 (63.3)	120 (55.8)	118 (55.4)	147 (68.4)
Former smoker	57 (26.8)	63 (28.9)	44 (20.8)	53 (24.7)	53 (24.9)	58 (27.0)	56 (26.0)	62 (29.1)	46 (21.4)
Current smoker	34 (16.0)	35 (16.1)	25 (11.8)	42 (19.5)	31 (14.6)	21 (9.8)	39 (18.1)	33 (15.5)	22 (10.2)
Missing	1 (0.5)	1 (0.46)	2 (0.93)	0 (0)	3 (1.4)	1 (0.5)	0 (0)	3 (1.39)	1 (0.46)
Food insecurity, n (%)									
No	123 (75.0)	137 (79.7)	136 (87.2)	125 (78.1)	121 (78.6)	150 (84.3)	134 (82.2)	125 (78.1)	137 (81.1)
Yes	41 (25.0)	35 (20.3)	20 (12.8)	35 (21.9)	33 (21.4)	28 (15.7)	29 (17.8)	35 (21.9)	32 (18.9)
Missing	50 (23.4)	47 (21.5)	58 (27.1)	55 (25.6)	62 (28.7)	38 (17.6)	52 (24.2)	56 (25.9)	47 (21.8)
BMI, mean (SD), kg/m^2^	30.8 (8.3)	31.1 (7.8)	30.3 (7.7)	31.6 (8.5)	31.2 (8.6)	29.3 (6.3)	31.6 (8.5)	30.5 (7.9)	30.1 (7.4)
Missing	1 (0.5)	0 (0)	0 (0)	0 (0)	1 (0.5)	0 (0)	1 (0.47)	0 (0)	0 (0)
BMI, n (%)									
Normal weight	55 (25.8)	48 (21.9)	53 (24.8)	48 (22.3)	51 (23.7)	57 (26.4)	45 (21.0)	57 (26.4)	54 (25.0)
Overweight	57 (26.8)	71 (32.4)	68 (31.8)	56 (26.0)	64 (29.8)	76 (35.2)	57 (26.6)	62 (28.7)	77 (35.6)
Obese	101 (47.4)	100 (45.7)	93 (43.5)	111 (51.6)	100 (46.5)	83 (38.4)	112 (52.3)	97 (44.9)	85 (39.4)
Missing	1 (0.5)	0 (0)	0 (0)	0 (0)	1 (0.5)	0 (0)	1 (0.5)	0 (0)	0 (0)
Modified Charlson CI, n (%)									
0	177 (82.7)	179 (81.7)	175 (81.8)	171 (79.5)	181 (83.8)	179 (82.9)	177 (82.3)	184 (85.2)	170 (78.7)
1	28 (13.1)	29 (13.2)	32 (15.0)	33 (15.3)	27 (12.5)	29 (13.4)	26 (12.1)	27 (12.5)	36 (16.7)
>=2	9 (4.2)	11 (5.0)	7 (3.3)	11 (5.1)	8 (3.7)	8 (3.7)	12 (5.6)	5 (2.3)	10 (4.6)
AP etiology, n (%)									
Gallstones	70 (32.9)	78 (37.1)	82 (39.0)	74 (34.7)	77 (36.5)	79 (37.8)	71 (33.2)	74 (35.6)	85 (40.3)
Alcohol	56 (26.3)	44 (21.0)	33 (15.7)	44 (20.7)	48 (22.7)	41 (19.6)	55 (25.7)	50 (24.0)	28 (13.3)
Others	87 (40.8)	88 (41.9)	95 (45.2)	95 (44.6)	86 (40.8)	89 (42.6)	88 (41.1)	84 (40.4)	98 (46.4)
Missing	1 (0.5)	9 (4.1)	4 (1.9)	2 (0.9)	5 (2.3)	7 (3.2)	1 (0.5)	8 (3.7)	5 (2.3)
History of prior AP, n (%)									
No	139 (65.3)	145 (66.5)	147 (69.7)	134 (63.2)	146 (67.6)	151 (70.6)	138 (65.1)	149(69.3)	144 (67.0)
Yes	74 (34.7)	73 (33.5)	64 (30.3)	78 (36.8)	70 (32.4)	63 (29.4)	74 (34.9)	66 (30.7)	71 (33.0)
Missing	1 (0.5)	1 (0.5)	3 (1.4)	3 (1.4)	0 (0)	2 (0.9)	3 (1.4)	1 (0.5)	1 (0.5)
Severity of AP, n (%)									
Mild	182 (85.4)	186 (86.1)	182 (85.4)	181 (84.2)	186 (87.7)	183 (85.1)	184 (85.6)	179 (84.4)	187 (87.0)
Moderate	26 (12.2)	20 (9.3)	24 (11.3)	26 (12.1)	18 (8.5)	26 (12.1)	24 (11.2)	26 (12.3)	20 (9.3)
Severe	5 (2.3)	10 (4.6)	7 (3.3)	8 (3.7)	8 (3.8)	6 (2.8)	7 (3.3)	7 (3.3)	8 (3.7)
Missing	1 (0.5)	3 (1.37)	1 (0.5)	0 (0)	4 (1.9)	1 (0.5)	0 (0)	4 (1.9)	1 (0.5)
MAFLD, n (%)									
No	173 (80.8)	186 (84.9)	180 (84.1)	165 (76.7)	191 (88.4)	183 (84.7)	170 (79.1)	187 (86.6)	182 (84.3)
Yes	41 (19.2)	33 (15.1)	34 (15.9)	50 (23.3)	25 (11.6)	33 (15.3)	45 (20.9)	29 (13.4)	34 (15.7)
Hypertension, n (%)									
No	116 (54.2)	137 (62.6)	131 (61.2)	124 (57.7)	123 (56.9)	137 (63.4)	116 (54.0)	134 (62.0)	134 (62.0)
Yes	98 (45.8)	82 (37.4)	83 (38.8)	91 (42.3)	93 (43.1)	79 (36.6)	99 (46.0)	82 (38.0)	82 (38.0)
Hyperlipidemia, n (%)									
No	151 (70.6)	154 (70.3)	154 (72.0)	142 (66.0)	158 (73.1)	159 (73.6)	149 (69.3)	159 (73.6)	151 (69.9)
Yes	63 (29.4)	65 (29.7)	60 (28.0)	73 (34.0)	58 (26.9)	57 (26.4)	66 (30.7)	57 (26.4)	65 (30.1)
Pre-existing diabetes, n (%)									
No	158 (73.8)	181 (82.6)	181 (84.6)	162 (75.3)	175 (81.0)	183 (84.7)	169 (78.6)	175 (81.0)	176 (81.5)
Yes	56 (26.2)	38 (17.4)	33 (15.4)	53 (24.7)	41 (19.0)	33 (15.3)	46 (21.4)	41 (19.0)	40 (18.5)
Total calories (kcal), mean (SD)	1891.1 (686.5)	1798.8 (769.2)	1865.3 (876.5)	2187.4 (785.6)	1664.7 (698.1)	1703.5 (749.0)	2373.7 (791.1)	1785.6 (602.1)	1397.1 (602.3)

Abbreviations: PDI: Plant-based Diet; rEDIP: reversed Empirical Dietary Inflammatory Pattern; rEDIH: reversed Empirical Dietary Index for Hyperinsulinemia; BMI: Body Mass Index; AP: Acute Pancreatitis; Charlson CI: Charlson Comorbidity Index; MAFLD: Metabolic dysfunction-associated fatty liver disease. Mean (SD) for continuous variables and proportion for categorical variables.

**Table 2:** Multivariable logistic regression results showing the ORs (95% CI) for pre-existing diabetes in DREAM participants with AP (n = 647) according to the dietary pattern scores (PDI, rEDIP, and rEDIH). Cases represent individuals with diabetes before the diagnosis of the AP episode who met the study inclusion criteria.

Dietary Patterns & Models	Tertile 1	Tertile 2	Tertile 3	Per 1-SD Increment	*p* Trend
PDI					
Cases/non-cases	56/158	38/181	33/181	127/520	
Median score (range)	53.80 (42.88, 57.19)	60.01 (57.19, 62.72)	65.94 (62.72, 79.98)	60.01 (42.88, 79.98)	
PDI-Model 1	1.00 (ref)	0.54 (0.33, 0.88)	0.48 (0.29, 0.79)	0.95 (0.92, 0.98)	0.003
PDI-Model 2	1.00 (ref)	0.54 (0.32, 0.91)	0.47 (0.27, 0.80)	0.94 (0.91, 0.98)	0.004
PDI-Model 3	1.00 (ref)	0.47 (0.27, 0.82)	0.45 (0.25, 0.81)	0.94 (0.90, 0.98)	0.005
PDI-Model 4	1.00 (ref)	0.46 (0.26, 0.81)	0.45 (0.25, 0.80)	0.94 (0.90, 0.98)	0.005
rEDIP					
Cases/non-cases	53/162	41/175	33/183	127/520	
Median score (range)	−0.95 (−3.31, −0.57)	−0.30 (−0.57, −0.06)	0.20 (−0.06, 2.79)	−0.30 (−3.31, 2.79)	
rEDIP-Model 1	1.00 (ref)	0.75 (0.46, 1.22)	0.56 (0.34, 0.93)	0.78 (0.59, 1.02)	0.023
rEDIP-Model 2	1.00 (ref)	0.75 (0.44, 1.26)	0.63 (0.37, 1.07)	0.80 (0.60, 1.07)	0.083
rEDIP-Model 3	1.00 (ref)	0.74 (0.42, 1.30)	0.66 (0.37, 1.17)	0.83 (0.61, 1.13)	0.139
rEDIP-Model 4	1.00 (ref)	0.76 (0.43, 1.33)	0.69 (0.39, 1.23)	0.84 (0.62, 1.15)	0.196
rEDIH					
Cases/non-cases	46/169	41/175	40/176	127/520	
Median score (range)	−1.75 (−4.74, −1.23)	−0.93 (−1.23, −0.65)	−0.32 (−0.65, 0.99)	−0.93 (−4.75, 0.99)	
rEDIH-Model 1	1.00 (ref)	0.88 (0.54, 1.45)	0.83 (0.50, 1.37)	0.85 (0.67, 1.08)	0.451
rEDIH-Model 2	1.00 (ref)	1.01 (0.59, 1.71)	0.88 (0.51, 1.50)	0.85 (0.66, 1.09)	0.658
rEDIH-Model 3	1.00 (ref)	1.06 (0.60, 1.86)	0.88 (0.49, 1.56)	0.85 (0.65, 1.11)	0.696
rEDIH-Model 4	1.00 (ref)	1.08 (0.61, 1.91)	0.92 (0.51, 1.64)	0.87 (0.66, 1.14)	0.814

Abbreviations: PDI: Plant-based Diet; rEDIP: reversed Empirical Dietary Inflammatory Pattern; rEDIH: reversed Empirical Dietary Index for Hyperinsulinemia. Model 1: Adjusted for age, sex, race, and total energy. Model 2: Adjusted for Model 1 covariates plus modified Charlson Comorbidity Index, hyperlipidemia, hypertension, and MAFLD. Model 3: Adjusted for Model 2 covariates plus smoking status, history of prior acute AP, etiology of AP, severity of AP, and time from AP diagnosis to FFQ completion. Model 4: Adjusted for Model 3 covariates plus BMI.

## Data Availability

Data described in the manuscript, codebook, and analytic code will not be made available because enrollment in the DREAM study is ongoing across 10 Clinical Centers. Upon completion of the study, data will be shared in accordance with NIH data-sharing policies.

## Supplementary Materials

The following supporting information can be downloaded at https://www.mdpi.com/article/10.3390/nu18213676/s1, Figure S1: Heatmap of Pearson correlations among dietary scores and BMI. Table S1: Food items included in the 18 food groups used to calculate plant-based dietary indices. Table S2: Food group components of the reversed empirical dietary index for hyperinsulinemia (rEDIH) and empirical dietary inflammatory pattern (rEDIP) scores. Table S3: Characteristics of DREAM participants according to tertiles of the hPDI and uPDI scores. Higher scores indicate greater adherence to diet patterns. Table S4: Multivariable logistic regression results showing the ORs (95% CI) for pre-existing diabetes in DREAM participants with AP (n = 647) according to the dietary pattern scores (hPDI and uPDI). Cases represent individuals with diabetes before the diagnosis of the AP episode who met the study inclusion criteria. Table S5: Sensitivity analysis of the associations between dietary pattern scores (PDI, rEDIP, rEDIH, hPDI, and uPDI) and pre-existing diabetes among DREAM participants without a history of AP prior to enrollment (n = 430). Table S6: Sensitivity analysis of the associations between dietary pattern scores (PDI, rEDIP, rEDIH, and uPDI) and pre-existing diabetes among DREAM participants (n = 647) using imputed missing covariate data.

## Abbreviations

The following abbreviations are used in this manuscript:

AP	Acute Pancreatitis
MIND	Mediterranean-DASH Intervention for Neurodegenerative Delay
EDIH	Empirical Dietary Index for Hyperinsulinemia
DGA	Dietary Guidelines for Americans
EDIP	Empirical Dietary Inflammatory Pattern
IL-6	Interleukin-6
CRP	*C*-reactive protein
TNFαR2	Tumor necrosis factor-α receptor 2
HbA1c	Hemoglobin A1c
BMI	Body mass index
SD	Standard deviation
OR	Odds Ratio
95% CI	95% Confidence intervals
U.S.	United States
ARIC	Atherosclerosis Risk in Communities
DNA	deoxyribonucleic acid
DREAM	Diabetes RElated to Acute Pancreatitis and its Mechanisms
rEDIH	reverse Empirical Dietary Index for Hyperinsulinemia
rEDIP	reverse Empirical Dietary Inflammatory Pattern
PDI	Plant-Based Diet
hPDI	Healthful Plant-Based Diet
uPDI	Unhealthful Plant Based
T1DAPC	Type 1 Diabetes in Acute Pancreatitis Consortium
NDSR	Nutrition Data System for Research
MAFLD	Metabolic dysfunction associated with fatty liver disease
CCI	Charlson Comorbidity Index
FFQ	Food Frequency Questionnaire
