# Supplementary material for: Inflammatory, Insulinemic, and Plant-Based Dietary Patterns and Diabetes Among Patients with Acute Pancreatitis: A Cross-Sectional Analysis of the DREAM Study
Source: Nutrients. 2026 Nov 6;18(21):3676. doi: 10.3390/nu18213676 (PMC13765010; doi:10.3390/nu18213676)

Supplemental Table S1. Food items included in the 18 food groups used to calculate plant-based dietary indices

Plant Food Groups	
Healthy	
**Whole grains**	Whole grain breakfast cereal, other cooked breakfast cereal, cooked oatmeal, dark bread, brown rice, other grains, bran, wheat germ, popcorn
**Fruits**	Raisins or grapes, prunes, bananas, cantaloupe, watermelon, fresh apples or pears, oranges, grapefruit, strawberries, blueberries, peaches or apricots or plums
**Vegetables**	Tomatoes, tomato juice, tomato sauce, broccoli, cabbage, cauliflower, Brussels sprouts, carrots, mixed vegetables, yellow or winter squash, eggplant or zucchini, yams or sweet potatoes, spinach cooked, spinach raw, kale or mustard or chard greens, iceberg or head lettuce, romaine or leaf lettuce, celery, mushrooms, beets, alfalfa sprouts, garlic, corn
**Nuts**	Nuts, peanut butter
**Legumes**	String beans, tofu or soybeans, beans or lentils, peas or lima beans
**Vegetable oils**	Oil-based salad dressing, vegetable oil used for cooking
**Tea & Coffee**	Tea, coffee, decaffeinated coffee
Less healthy	
**Fruit juices**	Apple cider (non-alcoholic) or juice, orange juice, grapefruit juice, other fruit juice
**Refined grains**	Refined grain breakfast cereal, white bread, English muffins or bagels or rolls, muffins or biscuits, white rice, pancakes or waffles, crackers, pasta
**Potatoes**	French fries, baked or mashed potatoes, potato or corn chips
**Sugar sweetened beverages**	Colas with caffeine & sugar, colas without caffeine but with sugar, other carbonated beverages with sugar, non-carbonated fruit drinks with sugar
**Sweets and Desserts**	Chocolates, candy bars, candy without chocolate, cookies (home-baked & ready-made), brownies, doughnuts, cake (home-baked & ready-made), sweet roll (home-baked & ready-made), pie (home-baked & ready-made), jams or jellies or preserves or syrup or honey
Animal Food Groups	
**Animal fat**	Butter added to food, butter or lard used for cooking
**Dairy**	Skim low fat milk, whole milk, cream, sour cream, sherbet, ice cream, yogurt, cottage or ricotta cheese, cream cheese, other cheese
**Egg**	Eggs
**Fish or Seafood**	Canned tuna, dark meat fish, other fish, shrimp or lobster or scallops
**Meat**	Chicken or turkey with skin, chicken or turkey without skin, bacon, hot dogs, processed meats, liver, hamburger, beef or pork or lamb mixed dish, beef or pork or lamb main dish
**Misc. animal-based foods**	Pizza, chowder or cream soup, mayonnaise or other creamy salad dressing

Supplemental Table S2. Food group components of the reversed empirical dietary index for hyperinsulinemia (rEDIH) and empirical dietary inflammatory pattern (rEDIP) scores.

rEDIH components[a]	Weight	Food items
		Food components positively associated with c-peptide concentrations
Processed meat	-0.199	Processed meats (lunch meat other lunch meat), bacon, hot dog
Red meat	-0.25	Beef, pork and lamb as a main dish, ground meat including hamburgers, Beef, pork, and lamb as a sandwich, stew, pot pie and casseroles with meat, gravies made with meat drippings, Menudo and tortilla soup
High-energy sugary beverages	-0.104	Regular soft drinks (not diet)
Margarine	-0.054	Margarine
Butter	-0.094	Butter
French fries	-0.581	French fries
Non-dark fish	-0.172	Tuna, shrimp, lobster, scallops, seafood other than dark fish
Eggs	-0.124	Egg
Low-fat dairy	-0.025	Low-fat milk, sherbet or ice milk, yogurt, low-fat desserts
Cream soup	-0.787	Chowder or cream soup
Tomatoes	-0.095	Fresh tomato & tomato juice, tomato sauce
Poultry	-0.183	Chicken & turkey, fried chicken, Chicken or turkey with or without skin
		Food components inversely associated with c-peptide concentrations
Green leafy vegetables	0.055	Spinach& mustard greens& turnip greens& collards, iceberg or head lettuce, romaine or leaf lettuce
Wine	0.165	Red, white wine
Coffee	0.035	Coffee (regular or decaffeinated)
High-fat diary	0.046	Whole milk, cream, sour cream ice cream, cream cheese, other cheese
Whole fruit	0.029	Raisins, grapes, avocado, banana, cantaloupe, watermelon, orange, apple, pear, grapefruit, strawberries, blueberries, peaches, apricots, plums

rEDIP components[b]	Weight	Food items
		Food components positively associated with proinflammatory biomarkers
Processed meat	-157.12082	Hot dogs, processed meats (including processed meat sandwich) , bacon
Red meat	-135.78592	Hamburger, beef /pork /lamb sandwich, beef /pork/ lamb main dish
Organ meat	-45.52817	Livers
Other fish	-243.82936	Canned tuna, shrimp, breaded fish, lobster, scallops or other seafood
Other vegetables	-136.89117	Corn, mixed vegetables, eggplant, celery, alfalfa sprouts, mushrooms, green/yellow/red peppers, zucchini, cucumbers
Refined grain	-87.0247	White bread, white rice, bagels/English muffins/rolls, muffins or biscuits, pasta, pancakes or waffles, refined cold breakfast cereals
High energy beverage	-154.80026	Cola, Hawaiian punch, caffeine-free Coke, Pepsi, carbonated beverage with caffeine and sugar, other carbonated beverage with sugar
Tomato	-160.65882	Fresh tomatoes, tomato juice, tomato sauce
		Food components inversely associated with proinflammatory biomarkers
Beer	135.2397	Beer, light beer
Wine	248.8155	White wine, red wine
Tea	128.297	Tea, tea (not herbal)
Coffee	128.297	Coffee, decaffeinated coffee
Dark yellow vegetable	166.1962	Carrots, sweet potatoes, winter squash
Green leafy vegetable	188.9353	Spinach, iceberg lettuce, romaine lettuce
Snack	43.82505	Potato/corn chips, popcorn, crackers
Fruit juice	60.66021	Apple juice, orange juice, grape juice, prune juice, other juice
Pizza	1169.052	Pizza

Supplemental Table S3. Characteristics of DREAM participants according to tertiles of the hPDI, and uPDI scores. Higher scores indicate greater adherence to diet patterns.

Characteristics	hPDI			uPDI		
	Tertile 1<br>N = 214	Tertile 2<br>N = 219	Tertile 3<br>N = 214	Tertile 1<br>N = 214	Tertile 2<br>N = 219	Tertile 3<br>N = 214
Scores, median (range)	52.7<br>(36.5, 56.4)	59.4<br>(56.5, 63.1)	67.5<br>(63.2, 84.3)	52.3<br>(41.0, 56.4)	60.0<br>(56.4, 63.4)	67.5<br>(63.4, 88.7)
Age, mean (SD), years	49.3 (14.8)	49.0 (14.6)	48.9 (14.2)	48.8 (13.8)	49.9 (15.1)	48.4 (14.6)
Age group, n (%)						
18-30	25 (11.7)	28 (12.8)	24 (11.2)	22 (10.3)	29 (13.2)	26 (12.1)
31-60	128 (59.8)	131 (59.8)	139 (65.0)	141 (65.9)	126 (57.5)	131 (61.2)
61-75	61 (28.5)	60 (27.4)	51 (23.8)	51 (23.8)	64 (29.2)	57 (26.6)
Sex, n (%)						
Male	100 (46.7)	103 (47.0)	94 (43.9)	90 (42.1)	119 (54.3)	88 (41.1)
Female	114 (53.3)	116 (53.0)	120 (56.1)	124 (57.9)	100 (45.7)	126 (58.9)
Race, n (%)						
White/Caucasian	159 (74.3)	173 (79.7)	159 (75.7)	166 (78.7)	174 (79.8)	151 (71.2)
Black	42 (19.6)	24 (11.1)	30 (14.3)	21 (10.0)	28 (12.8)	47 (22.2)
Others, multiple	13 (6.1)	20 (9.2)	21 (10.0)	24 (11.4)	16 (7.3)	14 (6.6)
Missing	0 (0.0)	2 (0.91)	4 (1.87)	3 (1.4)	1 (0.5)	2 (0.9)
Education, n (%)						
Less than high school	4 (2.6)	11 (6.1)	8 (5.3)	2 (1.3)	13 (7.6)	8 (5.1)
High school graduate or equivalent	32 (20.6)	40 (22.1)	25 (16.4)	23 (14.4)	33 (19.3)	41 (26.1)
Some college or associate degree	58 (37.4)	56 (30.9)	48 (31.6)	56 (35.0)	46 (26.9)	60 (38.2)
College graduate or higher	61 (39.4)	74 (40.9)	71 (46.7)	79 (49.4)	79 (46.2)	48 (30.6)
Missing	59 (27.6)	38 (17.4)	62 (29.0)	54 (25.2)	48 (21.9)	57 (26.6)
Annual income, n (%)						
Under $50,000	41 (31.8)	42 (26.4)	46 (34.3)	37 (25.0)	49 (32.9)	43 (34.4)
$50,000-$99,999	38 (29.5)	46 (28.9)	29 (21.6)	40 (27.0)	39 (26.2)	34 (27.2)
Over $100,000	50 (38.8)	71 (44.7)	59 (44.0)	71 (48.0)	61 (40.9)	48 (38.4)
Missing	85 (39.7)	60 (27.4)	80 (37.4)	66 (30.8)	70 (32.0)	89 (41.6)
Employment, n (%)						

Employed	99 (64.7)	117 (65.7)	90 (60.4)	103 (64.4)	104 (63.0)	99 (63.9)
Unemployed for other reasons	39 (25.5)	47 (26.4)	45 (30.2)	43 (26.9)	53 (32.1)	35 (22.6)
Unemployed for medical reasons	15 (9.8)	14 (7.9)	14 (9.4)	14 (8.8)	8 (4.8)	21 (13.5)
Missing	61 (28.5)	41 (18.7)	65 (30.4)	54 (25.2)	54 (24.7)	59 (27.6)
Smoking status, n (%)						
Never smoker	120 (56.3)	134 (61.5)	131 (61.8)	125 (59.2)	140 (63.9)	120 (56.3)
Former smoker	56 (26.3)	56 (25.7)	52 (24.5)	65 (30.8)	49 (22.4)	50 (23.5)
Current smoker	37 (17.4)	28 (12.8)	29 (13.7)	21 (10.0)	30 (13.7)	43 (20.2)
Missing	1 (0.5)	1 (0.5)	2 (0.9)	3 (1.4)	0 (0)	1 (0.5)
Food insecurity, n (%)						
No	120 (75.9)	149 (81.9)	127 (83.6)	138 (84.7)	138 (80.2)	120 (76.4)
Yes	38 (24.1)	33 (18.1)	25 (16.4)	25 (15.3)	34 (19.8)	37 (23.6)
Missing	56 (26.2)	37 (16.9)	62 (29.0)	51 (23.8)	47 (21.5)	57 (26.6)
BMI, mean (SD), kg/m^2	32.5 (8.9)	30.5 (7.6)	29.2 (6.9)	29.4 (7.2)	31.0 (7.5)	31.8 (8.9)
Missing	1 (0.5)	0 (0)	0 (0)	0 (0)	0 (0)	1 (0.5)
BMI, n (%)						
Normal weight	43 (20.2)	52 (23.7)	61 (28.5)	59 (27.6)	50 (22.8)	47 (22.1)
Overweight	53 (24.9)	70 (32.0)	73 (34.1)	71 (33.2)	70 (32.0)	55 (25.8)
Obese	117 (54.9)	97 (44.3)	80 (37.4)	84 (39.3)	99 (45.2)	111 (52.1)
Missing	1 (0.5)	0 (0)	0 (0)	0 (0)	0 (0)	1 (0.5)
Modified Charlson CI, n (%)						
0	171 (79.9)	182 (83.1)	178 (83.2)	183 (85.5)	175 (79.9)	173 (80.8)
1	30 (14.0)	30 (13.7)	29 (13.6)	23 (10.7)	35 (16.0)	31 (14.5)
>=2	13 (6.1)	7 (3.2)	7 (3.3)	8 (3.7)	9 (4.1)	10 (4.7)
AP etiology, n (%)						
Gallstones	79 (37.4)	73 (34.1)	78 (37.5)	66 (31.7)	84 (38.7)	80 (38.5)
Alcohol	50 (23.7)	41 (19.2)	42 (20.2)	49 (23.6)	37 (17.1)	47 (22.6)
Others	82 (38.9)	100 (46.7)	88 (42.3)	93 (44.7)	96 (44.2)	81 (38.9)
Missing	3 (1.4)	5 (2.3)	6 (2.8)	6 (2.8)	2 (0.9)	6 (2.8)
History of prior AP, n (%)						
No	145 (67.8)	139 (64.4)	147 (69.3)	145 (68.7)	150 (68.8)	136 (63.8)
Yes	69 (32.2)	77 (35.6)	65 (30.7)	66 (31.3)	68 (31.2)	77 (36.2)

Missing	0 (0)	3 (1.4)	2 (0.9)	3 (1.4)	1 (0.5)	1 (0.5)
Severity of AP, n (%)						
Mild	181 (85.4)	186 (85.7)	183 (85.9)	190 (89.6)	182 (83.5)	178 (84.0)
Moderate	23 (10.8)	23 (10.6)	24 (11.3)	19 (9.0)	30 (13.8)	21 (9.9)
Severe	8 (3.8)	8 (3.7)	6 (2.8)	3 (1.4)	6 (2.8)	13 (6.1)
Missing	2 (0.9)	2 (0.9)	1 (0.5)	2 (0.9)	1 (0.5)	2 (0.9)
MAFLD, n (%)						
No	174 (81.3)	185 (84.5)	180 (84.1)	177 (82.7)	185 (84.5)	177 (82.7)
Yes	40 (18.7)	34 (15.5)	34 (15.9)	37 (17.3)	34 (15.5)	37 (17.3)
Hypertension, n (%)						
No	114 (53.3)	139 (63.5)	131 (61.2)	126 (58.9)	134 (61.2)	124 (57.9)
Yes	100 (46.7)	80 (36.5)	83 (38.8)	88 (41.1)	85 (38.8)	90 (42.1)
Hyperlipidemia, n (%)						
No	155 (72.4)	162 (74.0)	142 (66.4)	158 (73.8)	148 (67.6)	153 (71.5)
Yes	59 (27.6)	57 (26.0)	72 (33.6)	56 (26.2)	71 (32.4)	61 (28.5)
Preexisting diabetes, n (%)						
No	168 (78.5)	173 (79.0)	179 (83.6)	177 (82.7)	167 (76.3)	176 (82.2)
Yes	46 (21.5)	46 (21.0)	35 (16.4)	37 (17.3)	52 (23.7)	38 (17.8)
Total calories (kcal), mean (SD)	1,864.3 (788.5)	1,779.1 (791.9)	1,912.2 (759.7)	1,906.2 (754.3)	1,741.1 (801.2)	1,909.3 (777.8)

Abbreviations: hPDI: Healthful Plant-based Diet; uPDI: Unhealthful Plant-based Diet; BMI: Body Mass Index; AP: Acute Pancreatitis; Charlson CI: Charlson Comorbidity Index; MAFLD: Metabolic dysfunction-associated fatty liver disease; FFQ: Food Frequency Questionnaire. Mean (SD) for continuous variables and proportion for categorical variables. Percentages for mom-missing categories were calculated among participants with available data, whereas percentages for missing data were calculated using the total number of participants in each tertile. Therefore, percentage may not sum to 100% across all categories.

Supplementary Table S4. Multivariable logistic regression results showing the ORs (95% CI) for preexisting diabetes in DREAM participants with AP (n=647) according to the dietary pattern scores (hPDI and uPDI). Cases represent individuals with diabetes before the diagnosis of the AP episode who met the study inclusion criteria.

Dietary Patterns & Models*	Tertile 1	Tertile 2	Tertile 3	Per 1-SD increment	*P* trend
hPDI†					
Cases /non-cases	46/168	46/173	35/179	127/520	
Median score (range)	52.66 (36.54, 56.40)	59.44 (56.46, 63.13)	67.46 (63.20, 84.27)	60 (38, 85)	
hPDI - Model 1	1.00 (ref)	1.074 (0.662, 1.741)	0.761 (0.458, 1.265)	0.997 (0.971, 1.024)	0.284
hPDI - Model 2	1.00 (ref)	1.208 (0.718, 2.032)	0.681 (0.394, 1.177)	0.993 (0.965, 1.022)	0.155
hPDI - Model 3	1.00 (ref)	1.203 (0.629, 2.092)	0.637 (0.356, 1.140)	0.990 (0.960, 1.020)	0.123
hPDI - Model 4	1.00 (ref)	1.232 (0.707, 2.149)	0.675 (0.374, 1.219)	0.993 (0.963, 1.024)	0.187
UPDI †					
Cases/non-cases	37/177	52/167	38/176	127/520	
Median score (range)	52.28 (41.01, 56.36)	59.99 (56.40, 63.40)	67.45 (63.40, 88.66)	60 (42, 89)	
uPDI - Model 1	1.00 (ref)	1.348 (0.824, 2.205)	0.916 (0.543, 1.547)	1.003 (0.977, 1.031)	0.754
uPDI - Model 2	1.00 (ref)	1.326 (0.784, 2.242)	0.856 (0.491, 1.492)	1.004 (0.975, 1.033)	0.592
uPDI - Model 3	1.00 (ref)	1.278 (0.729, 2.243)	0.842 (0.464, 1.528)	1.001 (0.970, 1.033)	0.565
uPDI - Model 4	1.00 (ref)	1.237 (0.702, 2.179)	0.807 (0.442, 1.472)	0.999 (0.967, 1.031)	0.474

Abbreviations: hPDI: Healthful Plant-based Diet; uPDI: Unhealthful Plant-based Diet. Model 1: Adjusted for age, sex, race, and total energy. Model 2: Adjusted for Model 1 covariates plus modified Charlson Comorbidity Index, hyperlipidemia, hypertension, and MAFLD. Model 3: Adjusted for Model 2 covariates plus smoking status, history of prior acute AP, etiology of AP, severity of AP, and time from AP diagnosis to FFQ completion. Model 4: Adjusted for Model 3 covariates plus BMI.

Supplementary Table S5. Sensitivity analysis of the associations between dietary pattern scores (PDI, rEDIP, rEDIH, hPDI, and uPDI) and pre-existing diabetes among DREAM participants without a history of AP prior to enrollment (n = 430).

Dietary Patterns	Tertile 1	Tertile 2	Tertile 3	Per 1-SD increment	*P* trend
PDI†	1.00 (ref)	0.529 (0.254, 1.106)	0.445 (0.209, 0.944)	0.955 (0.907, 1.005)	0.033
rEDIP	1.00 (ref)	1.002 (0.479, 2.095)	0.692 (0.322, 1.488)	0.916 (0.598, 1.405)	0.374
rEDIH	1.00 (ref)	1.605 (0.481, 3.517)	1.056 (0.481, 2.320)	0.978 (0.679, 1.408)	0.843
hPDI†	1.00 (ref)	1.002 (0.476, 2.110)	0.759 (0.357, 1.613)	0.996 (0.956, 1.038)	0.468
uPDI †	1.00 (ref)	1.105 (0.509, 2.403)	0.927 (0.412, 2.085)	1.018 (0.976, 1.063)	0.831

Abbreviations: PDI: Plant-based Diet; rEDIP: reversed Empirical Dietary Inflammatory Pattern; rEDIH: reversed Empirical Dietary Index for Hyperinsulinemia; hPDI: Healthful Plant-based Diet; uPDI: Unhealthful Plant-based Diet. In the sensitivity analysis, we adjusted the model for age, sex, race, total energy intake, Charlson Comorbidity Index, hyperlipidemia, hypertension, MAFLD, smoking status, AP etiology, AP severity, time from AP diagnosis to FFQ completion, and BMI.

Supplementary Table S6. Sensitivity analysis of the associations between dietary pattern scores (PDI, rEDIP, rEDIH, and uPDI) and pre-existing diabetes among DREAM participants (n = 647) using imputed missing covariate data.

Dietary Patterns	Tertile 1	Tertile 2	Tertile 3	Per 1-SD increment	*P* trend
PDI†	1.00 (ref)	0.481 (0.276, 0.839)	0.463 (0.261, 0.821)	0.938 (0.903, 0.976)	0.006
rEDIP	1.00 (ref)	0.734 (0.419, 1.285)	0.678 (0.382, 1.203)	0.839 (0.617, 1.140)	0.165
rEDIH	1.00 (ref)	1.050 (0.598, 1.845)	0.887 (0.499, 1.578)	1.024 (1.004, 1.045)	0.717
hPDI†	1.00 (ref)	1.268 (0.730, 2.204)	0.678 (0.376, 1.220)	0.993 (0.963, 1.024)	0.185
uPDI †	1.00 (ref)	1.243 (0.708, 2.183)	0.796 (0.440, 1.441)	0.998 (0.967, 1.030)	0.442

Abbreviations: PDI: Plant-based Diet; rEDIP: reversed Empirical Dietary Inflammatory Pattern; rEDIH: reversed Empirical Dietary Index for Hyperinsulinemia; hPDI: Healthful Plant-based Diet; uPDI: Unhealthful Plant-based Diet. The multivariable model was adjusted for age, sex, race, total energy intake, Charlson Comorbidity Index, hyperlipidemia, hypertension, MAFLD, smoking status, AP etiology, AP severity, time from AP diagnosis to FFQ completion, and BMI. We handled missing covariate values by assigning a separate "missing" category for categorical variables and imputing missing continuous values with the sample mean.

Supplementary Figure S1: Heatmap of Pearson correlations among dietary scores and BMI.

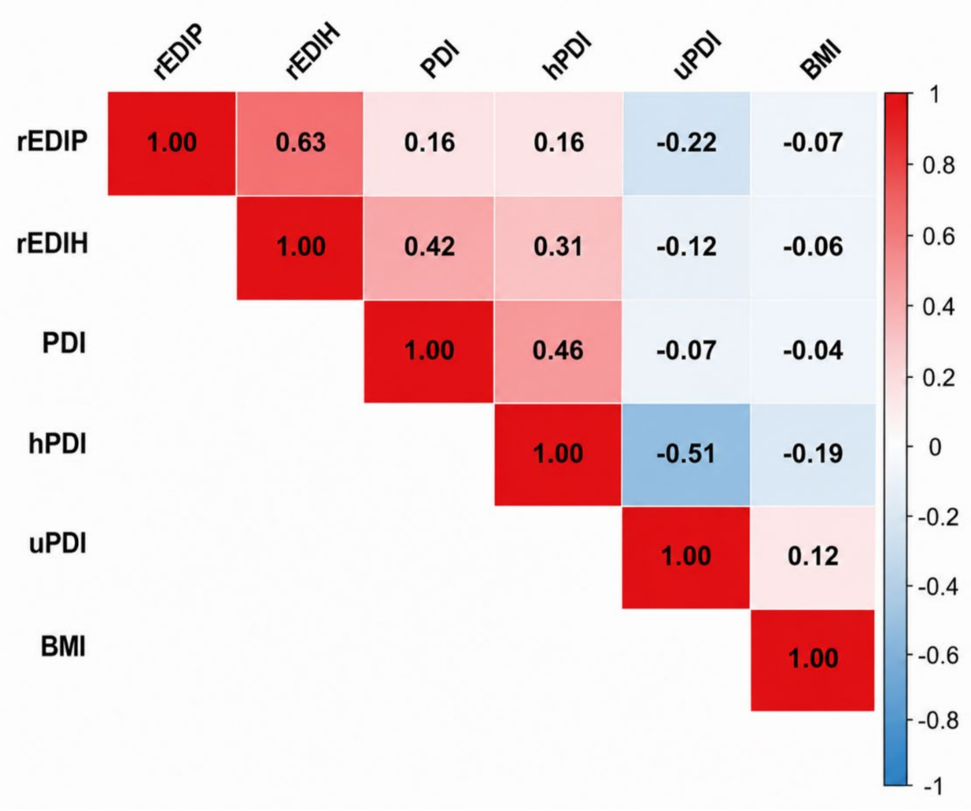

Supplement: Supplementary file 1 [file nutrients-18-03676-s001.zip › nutrients-4622124-supplementary.pdf]
